# Inhibiting Multiple Deubiquitinases to Reduce Androgen Receptor Expression in Prostate Cancer Cells

**DOI:** 10.1038/s41598-018-31567-3

**Published:** 2018-09-03

**Authors:** Alicia de las Pozas, Teresita Reiner, Virginia De Cesare, Matthias Trost, Carlos Perez-Stable

**Affiliations:** 1 Geriatric Research, Education, and Clinical Center and Research Service, Bruce W. Carter Veterans Affairs Medical Center, Miami, FL 33125 USA; 2 Division of Gerontology & Palliative Medicine, Department of Medicine, University of Miami Miller School of Medicine, Miami, FL 33136 USA; 3 Sylvester Comprehensive Cancer Center, University of Miami Miller School of Medicine, Miami, FL 33136 USA; 4 MRC Protein Phosphorylation & Ubiquitylation Unit, University of Dundee, Dundee, Scotland UK; 5 Institute for Cell and Molecular Biosciences, Newcastle University, Newcastle upon Tyne, UK

## Abstract

Prostate cancer (PCa), a leading cause of cancer-related death in men, becomes resistant to androgen deprivation therapy by inducing androgen receptor (AR) activity, which is known as castration-resistant PCa (CRPC). Enzalutamide is an approved drug that inhibits AR activity and increases overall survival. However, resistance to enzalutamide develops rapidly often by increasing AR activity, suggesting that new therapies are required for CRPC. We investigated whether betulinic acid (BA), a small molecule from plants that inhibits multiple deubiquitinases (DUBs), reduces AR, and selectively kills PCa cells, can provide an adjuvant strategy for CRPC. Our data indicated that BA reduced AR protein stability and mRNA expression, making it an attractive agent for CRPC. BA decreased AR mRNA possibly by inhibiting a histone 2A DUB thereby increasing ubiquitinated histone 2A, a transcriptional repressor. We identified multiple and specific DUBs inhibited by BA either in PCa cells or using recombinant DUBs. Similar results were obtained using another multi-DUB inhibitor WP1130, suggesting that these DUB inhibitors can decrease AR expression and increase PCa-specific death. Our results also suggest that combining multi-DUB inhibitors BA or WP1130 with enzalutamide may provide a novel strategy for CRPC by further decreasing AR expression and increasing apoptotic cell death.

## Introduction

Prostate cancer (PCa) is a leading cause of cancer-related death in men, especially when metastasis occurs. Although initially responsive to androgen deprivation therapy (ADT), PCa cells can adapt to grow in low androgen levels by inducing androgen receptor (AR) expression and signaling, which leads to the progression of castration-resistant PCa (CRPC)^1,2^. Because CRPC maintains a dependency on AR and androgens^3^, the development of new agents that antagonize AR signaling has resulted in increased overall survival. For example, enzalutamide (Enz) is a specific AR antagonist that increases overall PCa survival^4^. However, initial insensitivity or acquired resistance to Enz is a common occurrence, indicating that new therapies are required for CRPC^5^.

The strategy of discovering small molecule drugs to enhance protein degradation including AR has not been fully exploited as a therapeutic option in CRPC. We previously reported that the PCa-specific ability of betulinic acid (BA), a plant-derived small molecule, to decrease several pro-survival proteins including AR and increase cell death may be due to inhibition of multiple deubiquitinases (DUBs) in cancer but not in non-cancer cells^6–8^. Since resistance to Enz is a common occurrence in the clinic^5^, we hypothesize that adding a multi-DUB inhibitor such as BA to ADT may provide a powerful approach against CRPC by decreasing AR expression, increasing cell death, and possibly overcome resistance to Enz with minimal toxicity to normal cells.

Reversible ubiquitination (Ub) is a crucial mechanism in the regulation of the ubiquitin proteasome system (UPS) and the concentrations of many pro-survival proteins^9–11^. Recent findings indicate that DUBs have critical regulatory roles in most pathways involving Ub. There are approximately 100 human DUBs, the best characterized being Ub specific proteases (USP) and Ub C-terminal hydrolases (UCHL). DUBs increase the stability of key proteins by negatively regulating UPS-mediated degradation. Removal of poly-Ub from key proliferation and pro-survival proteins renders them less susceptible to degradation by the UPS and increases their levels. In fact, several DUBs are reported to be overexpressed in cancer and are characterized as oncogenes^9–11^.

Several studies suggest that DUBs are valid targets for treatment of PCa^12–15^. There is evidence that specific DUBs regulate AR protein stability and downstream signaling. For example, USP10 is an AR cofactor important for activation of AR regulated genes^16–18^ and USP26 can also influence AR activity and stability^19^. More recently, USP12, 22, 7, and 14 have been shown to regulate AR accumulation, signaling, and binding to the chromatin^20–23^. Because DUBs appear to have a role in oncogenic transformation^9–11^, recent attention has focused on the identification of small molecule inhibitors of DUBs^24–26^. The idea is that inhibiting DUBs will elevate poly-Ub on proliferation/pro-survival proteins, increase their recognition and degradation by the UPS, result in greater apoptosis, and improve drug efficacy. Several small molecule DUB inhibitors increase accumulation of poly-Ub proteins and result in greater apoptosis in cancer cells^27–32^. Currently, DUB inhibitors are in the preclinical stage of research and no results from clinical trials are yet known.

In this report, we focused on the ability of BA to reduce AR expression in PCa cells, which makes it an attractive anti-CRPC agent. Our results showed that BA decreased AR protein stability, which is dependent on an active UPS but not on AKT, ERK, or JNK signaling. BA also decreased AR mRNA, possibly due to increased Ub-histone 2A (Ub-H2A), a known epigenetic transcriptional repressor^33–35^. We identified several DUBs that are inhibited by BA using PCa cells or recombinant proteins. Another multi-DUB inhibitor, WP1130^27^, also reduced AR protein, increased poly-Ub/cell death, and decreased DUB activity in PCa but not in non-cancer prostate epithelial cells. Finally, we showed that the AR antagonist Enz combined with BA enhanced AR degradation and cell death in CRPC cells. Overall, our results suggest that small molecule multi-DUB inhibitors such as BA can be combined with Enz to efficiently reduce AR expression and increase apoptotic cell death of CRPC cells with less toxic side effects.

## Results

### BA enhances apoptotic and necrotic cell death in PCa cells

**Figure 1:**
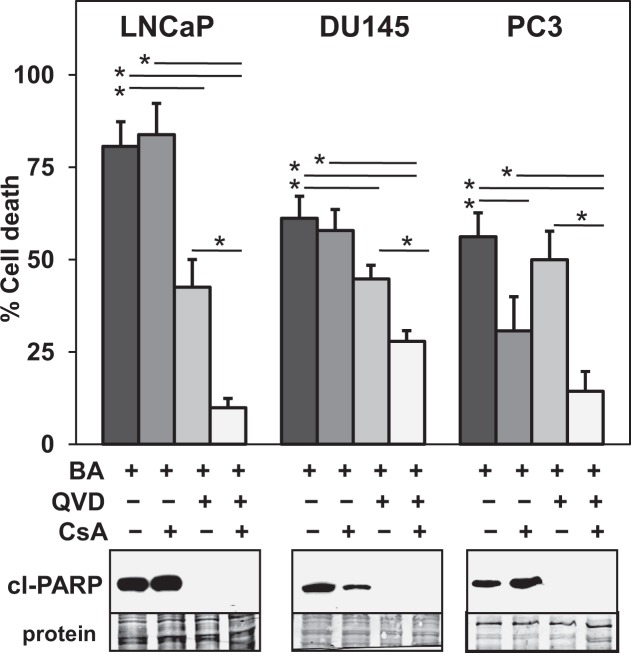
BA increases apoptotic and necrotic cell death in multiple PCa cell lines. Trypan blue exclusion assay (LNCaP 48 h and DU145, PC3 72 h) showed that CsA (10 μM; inhibits necrosis) reduced BA (10 μM) cell death only in PC3. Pan-caspase inhibitor QVD (10 μM; inhibits apoptosis) reduced BA cell death and cl-PARP (western blot) in LNCaP and DU145. QVD also reduced cl-PARP in PC3 but had no effect on BA cell death. Addition of CsA and QVD to BA reduced cell death in LNCaP, DU145, and PC3. Coomassie blue stain of total protein transferred to the membrane was the loading control for western blot. Blot images were cropped for clarity of the presentation. *P < 6 × 10^−3^; n = 6, 3 independent experiments.

BA is reported to target mitochondria and initiate the intrinsic pathway of apoptosis by increasing the release of mitochondrial proteins such as cytochrome c, which activates the caspase cascade^6,7^. BA is also reported to increase necrotic cell death via the mitochondrial permeability transport pore (MPTP) in a manner independent of pro-apoptotic Bax/Bak^36^. We determined in human PCa cell lines LNCaP, DU145, and PC3 the extent of apoptosis and necrosis dependent cell death after treatment with BA. Results indicated that the pan-caspase apoptosis inhibitor QVD reduced BA increase of cell death and cleaved (cl)-PARP (marker of apoptosis) in LNCaP and DU145. In contrast, QVD decreased cl-PARP but had no effect on cell death by BA in PC3 (Fig. 1). Cyclosporin A (CsA), known to bind to cyclophilin D, inhibit the MPTP, and block certain forms of necrotic cell death^37^, reduced BA cell death only in PC3 cells (Fig. 1). However, combining QVD and CsA further reduced BA cell death in LNCaP, DU145, and PC3. These results suggest that BA cell death in a variety of PCa cells is through caspase-dependent apoptotic and CsA-dependent necrotic pathways.

### BA reduces an AR variant associated with Enz resistance

**Figure 2:**
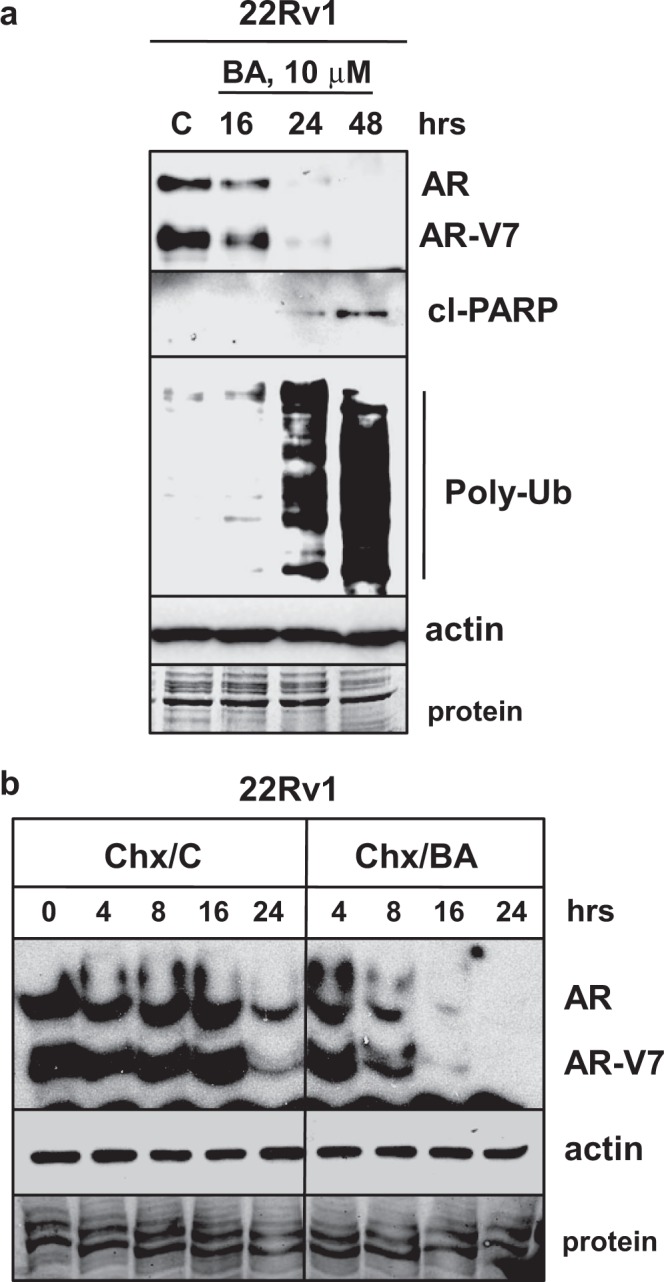
BA decreases AR variant associated with Enz resistance and lowers AR protein stability. (**a**) Western blot showed that BA (10 μM) decreased AR and AR-V7 variant and increased cl-PARP, poly-Ub without affecting actin in 22Rv1 cells. (**b**) Western blot showed that Chx (25 μg/ml) + BA (Chx/BA) decreased AR and AR-V7 protein more rapidly especially at 8, 16, and 24 h compared to Chx + control (Chx/C) in 22Rv1 cells. Actin was unaffected. Loading control (protein). Blot images were cropped for clarity of the presentation.

Treatment of LNCaP cells with BA decreases AR protein^8,38^. Because the progression of PCa to CRPC is mainly due to the reactivation of the AR signaling axis^1,2^, we focused our studies on the effects of BA on AR expression. AR mRNA splice variants without the C-terminal ligand binding domain are expressed in PCa clinical samples and cell lines and are important for the growth of CRPC and resistance to Enz^39–42^. 22Rv1 CRPC cells express high levels of AR-V7 variant, which is upregulated in patient samples of CRPC and confers a growth advantage in androgen-depleted conditions^39–43^. Similar to LNCaP, DU145, and PC3, cell proliferation assay indicated that 22Rv1 are sensitive to BA (Supplementary Fig. 1a). Our results confirmed that BA also reduced AR-V7 as well as full-length AR protein, increased cl-PARP, and poly-Ub in 22Rv1 cells (Fig. 2a). BA also increased poly-Ub in LNCaP, DU145, and PC3 (Supplementary Fig. 1b).

### BA-mediated AR protein degradation in LNCaP PCa cells is dependent on the UPS but not on AKT, ERK, or JNK signaling pathways

**Figure 3:**
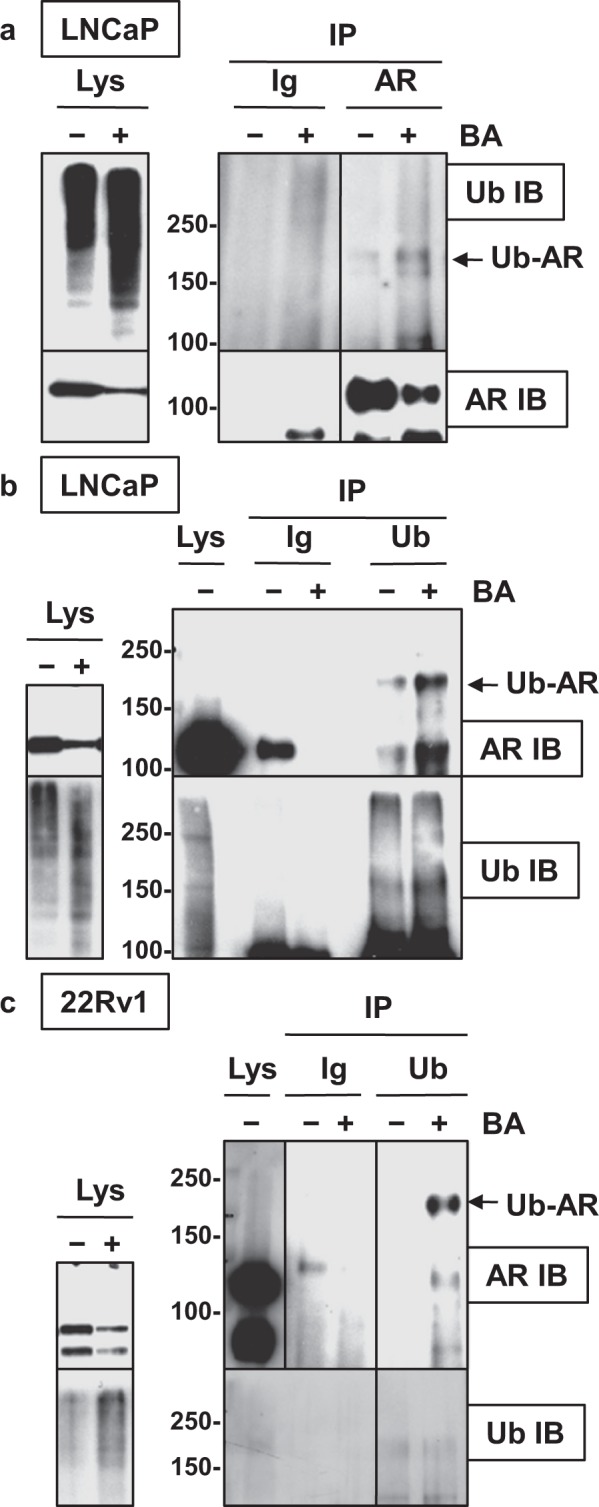
BA increases Ub-AR in LNCaP and 22Rv1. (**a**) Western blot showed that BA (10 μM; 20 h) increased Ub-AR (~200 kDa) compared to control in LNCaP after immunoprecipitation (IP) with AR and immunoblot (IB) with Ub. AR IP and AR IB showed the expected signal for AR. Negative control IP with mouse Ig showed no signal. Input lysate (Lys), shown to the left, was run on a separate gel. (**b**) Ub-AR also increased in BA compared to control LNCaP after Ub IP and AR IB. Ub IP and Ub IB showed the expected signal compared to negative control IP with mouse Ig. Total protein (Lys) was run on the same gel. Input Lys, shown to the left, was run on a separate gel. (**c**) In 22Rv1, Ub IP and AR IB also increased Ub-AR in BA compared to control. Ub IP and Ub IB showed the expected signal compared to negative control IP with mouse Ig. Total protein (Lys) was run on the same gel (exposure time for AR was less than IP samples). Input Lys, shown to the left, was run on a separate gel. Vertical line in (**a**,**c**) indicated samples were from the same blot but not in sequence. Molecular weight markers in kDa are shown to the left of IP/IB blots. Blot images were cropped for clarity of the presentation.

Evidence that BA reduces AR protein stability is indicated by our results with the cycloheximide (Chx) chase experiment. 22Rv1 cells were simultaneously treated with the protein synthesis inhibitor Chx + DMSO control (Chx/C) or BA (Chx/BA) and cells harvested at 0, 4, 8, 16, and 24 h. Western blot analysis showed that at 8, 16, and 24, Chx + BA reduced AR and AR-V7 protein greater than Chx + control (Fig. 2b). Similar results were obtained in LNCaP cells (Supplementary Fig. 2). It is likely that the decrease in AR protein in LNCaP and 22Rv1 with BA was due to elevated levels of Ub-AR, a substrate for protein degradation by the UPS (Fig. 3).

**Figure 4:**
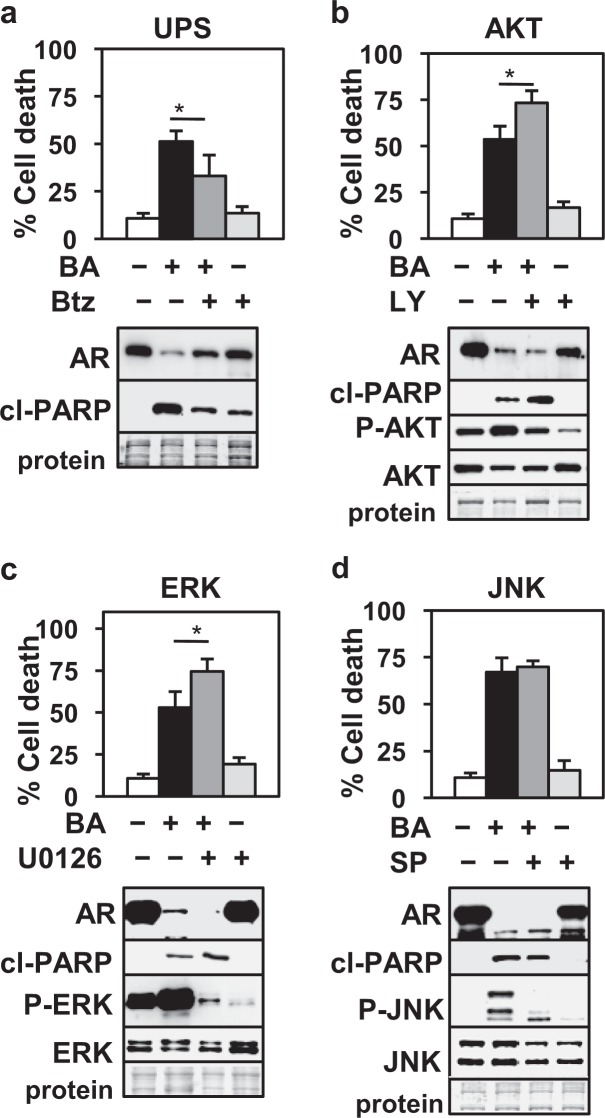
UPS but not AKT, ERK, or JNK inhibition blocks BA decrease of AR in LNCaP. Trypan blue and western blot showed that the (**a**) UPS inhibitor Btz (5 nM, 24 h) antagonized BA decrease in AR and increase in cell death, cl-PARP whereas (**b**) AKT inhibitor LY294002 (20 μM, 24 h), (**c**) ERK inhibitor U0126 (10 μM, 24 h), and (**d**) JNK inhibitor SP600125 (20 μM, 48 h) did not influence the BA decrease in AR or antagonize cell death/cl-PARP. AKT and ERK but not JNK inhibition further increased BA cell death. Activity of inhibitors was shown by blocking the BA increase in P-AKT, P-ERK, and P-JNK. There were no changes in total ERK or JNK; AKT decreased with BA. *P < 0.03); n = 6, 3 independent experiments. Loading control (protein). Blot images were cropped for clarity of the presentation.

To determine if AR protein degradation and apoptotic cell death with BA are dependent on the UPS pathway, LNCaP cells were treated with BA and the UPS inhibitor bortezomib (Btz). Results showed that Btz antagonized the BA decrease in AR and increase in cell death and cl- PARP (Fig. 4a). Since BA is known to have effects on multiple signaling pathways^44^, we investigated if inhibition of AKT, ERK, and JNK signaling pathways alters AR protein and apoptotic cell death. Treatment of LNCaP cells with BA increased phospho (P)-AKT, P-ERK, and P-JNK compared to control treated cells, suggesting that BA stimulated these signaling pathways (Fig. 4b–d). However, inhibition of AKT or ERK signaling had little effect on the BA decrease in AR protein and in fact increased BA cell death and cl-PARP (Fig. 4b,c). We suggest that the increase in AKT or ERK signaling is a pro-survival adaptation to BA treatment. Inhibition of JNK signaling had no effect on BA cell death, cl-PARP, or AR (Fig. 4d). Overall, these results suggest that despite BA having effects on multiple signaling pathways, the increase in the degradation of AR protein and in apoptotic cell death is dependent on an active UPS pathway.

### BA also decreases AR mRNA possibly by increasing Ub-H2A transcriptional repressor

**Figure 5:**
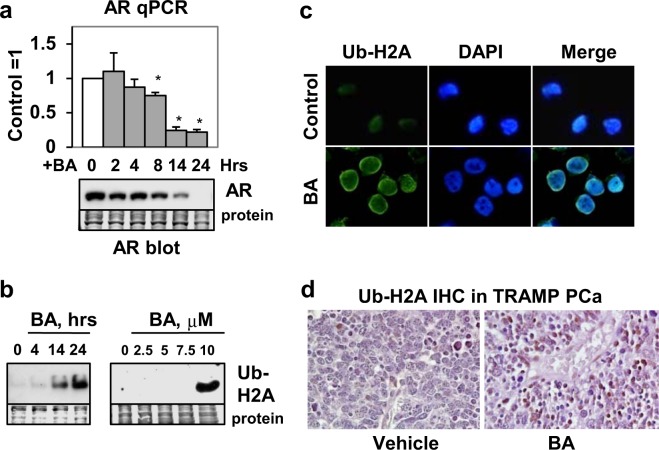
BA increases Ub-H2A transcriptional repressor and decreases AR mRNA. (**a**) qPCR showed that BA reduced AR mRNA (*P < 0.002; n = 4, 2 independent experiments) and protein (western blot) starting at 8 h in LNCaP. (**b**) Western blot in LNCaP showed that BA increased Ub-H2A (23 kDa) at 14 and 24 h (left blot); only the 10 μM concentration of BA known to inhibit DUB activity was able to increase Ub-H2A (24 h; right blot). Loading control (protein). Blot images in (**a**,**b**) were cropped for clarity of the presentation. (**c**) Immunofluorescence in LNCaP showed that BA (24 h) increased Ub-H2A (green) in the nucleus (DAPI stain, blue) greater than control cells. (**d**) IHC of PCa showed greater nuclear Ub-H2A (x200, dark brown color) in BA compared to vehicle control treated TRAMP mice.

Although previous studies show that BA decreases AR protein^8,38^, the effect of BA on AR mRNA expression is not known. LNCaP cells were treated with BA for 0, 2, 4, 8, 14, and 24 h and AR mRNA measured by real-time quantitative polymerase chain reaction (qPCR). The results demonstrated that BA reduced AR mRNA (and protein) starting at 8 h (Fig. 5a). Although one may conclude that the reason BA decreased AR protein is because of decreased AR mRNA, our previous results^8^ and the results in Figs 2b and 4a also suggest an effect on AR protein stability.

Because BA is proposed to inhibit multiple DUBs^8^, we investigated whether BA increased Ub-H2A protein, an epigenetic marker of repressed genes^33–35^. Western blot using anti-H2A-K119Ub showed that (1) BA increased Ub-H2A (23 kDa contains one Ub not poly-Ub) by 14 h, a time when AR mRNA is substantially decreased (Fig. 5a,b) and (2) only the concentration of BA that inhibits DUB activity (10 μM)^8^ increased Ub-H2A (Fig. 5b). Immunofluorescence of LNCaP cells after BA treatment showed an increase in nuclear Ub-H2A compared to control cells (Fig. 5c). Finally, immunohistochemistry (IHC) of PCa from TRAMP^45^ mice treated with BA showed higher expression of nuclear Ub-H2A compared to vehicle control tumors (Fig. 5d). Our previous IHC results demonstrates that BA reduces AR in TRAMP PCa but not in normal prostate^8^. These results suggest that the BA decrease in AR mRNA may be due to an increase in Ub-H2A transcriptional repressor.

### BA inhibits multiple DUBs in PCa cells

**Figure 6:**
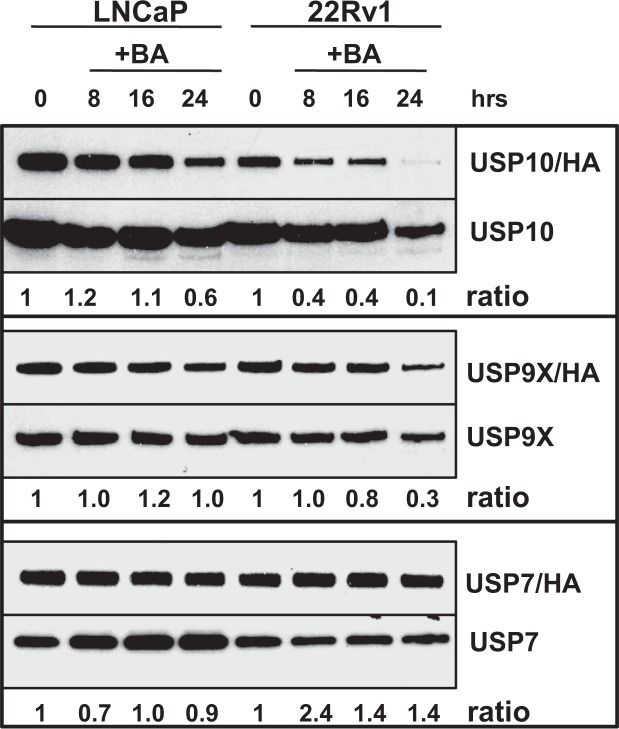
BA inhibits USP10 in LNCaP and 22Rv1. Western blot analysis of DUB labeling assay using HA-UbVS and USP10, 9X, and 7 specific antibodies. Results showed that BA inhibited USP10 activity (ratio of USP10/HA/total USP10 values shown below; 0 h = 1). BA inhibited USP9X activity in 22Rv1 but not in LNCaP. There was little effect of BA on USP7 activity. Blot images were cropped for clarity of the presentation. Similar results were obtained in an additional experiment.

We previously showed that BA inhibits multiple DUBs using a DUB labeling assay with HA-Ub vinyl sulfone (VS), a potent, irreversible, and specific inhibitor of most DUBs^8,46^. Since HA-UbVS only binds to active DUBs, the HA tag allows for the labeling of active DUBs present in PCa cells after BA treatment and analysis by western blot with anti-HA^8^. To begin to identify the specific DUBs inhibited by BA, we labeled LNCaP and 22Rv1 lysates with HA-UbVS after BA treatment and analyzed by western blot with a DUB-specific antibody (USP10, 9X, and 7). Standard LNCaP and 22Rv1 protein lysates without HA-UbVS labeling was also analyzed by western blot and the ratio of active USP/HA over total USP determined. Our results showed that BA treatment (1) decreased USP10 activity in both LNCaP and 22Rv1; (2) decreased USP9X activity in 22Rv1; and (3) had little effect on USP7 activity (Fig. 6).

### USP10 is a candidate DUB inhibited by BA that regulates AR protein and is expressed in human PCa tissues

**Figure 7:**
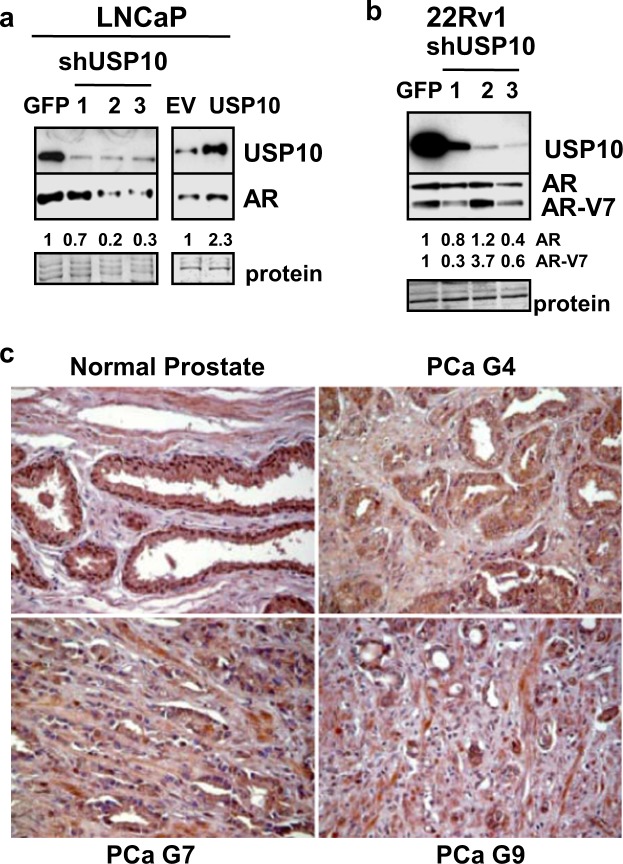
USP10, a candidate AR-regulatory DUB inhibited by BA, is variably expressed in human PCa tissues. (**a**) Western blot showed that stable knockdown (5- to 14-fold) of USP10 in LNCaP with 3 different shRNAs (shUSP10-1, -2, -3) reduced AR protein by 1.5- to 5-fold compared to shGFP control. Stable overexpression of USP10 (6-fold) increased AR protein 2-fold compared to empty vector (EV) control. Loading control (protein). (**b**) In 22Rv1, stable knockdown of USP10 (6- to 100-fold) reduced AR and AR-V7 protein by 1.5- to 3-fold in 2 of 3 shRNAs. Blot images in (**a**,**b**) were cropped for clarity of the presentation. (**c**) Representative IHC images of USP10 expression (x200, dark brown color) in human PCa tissues compared to normal prostate using a tissue microarray. Results showed that USP10 was highly expressed in cytoplasm and nucleus of epithelial cells in normal prostate. With higher Gleason (G7 and 9 compared to G4) grades of PCa, USP10 was more variably expressed with less nuclear localization.

To more specifically identify AR-regulatory DUBs inhibited by BA, we investigated USP10 because of its known role in AR signaling^16–18^. Stable knockdown of USP10 in LNCaP and 22Rv1 cells with 3 different shRNAs resulted in a 1.5 to 5-fold decrease in AR and AR-V7 protein compared to shGFP control cells (Fig. 7a,b). LNCaP cells overexpressing USP10 had ~2-fold greater AR protein compared to LNCaP/EV (empty vector) control cells (Fig. 7a).

To begin to determine if USP10 is expressed in human PCa, we obtained a PCa tissue microarray containing PCa categorized as Gleason grade 4–10 and normal prostate tissue. The initial IHC result showed that USP10 was strongly expressed in epithelial cells (cytoplasm and nucleus) of normal prostate (Fig. 7c). In PCa tissues, however, there was greater variability in USP10 expression and less nuclear localization with increasing Gleason grades (Fig. 7c).

### BA inhibits recombinant USP21

**Figure 8:**
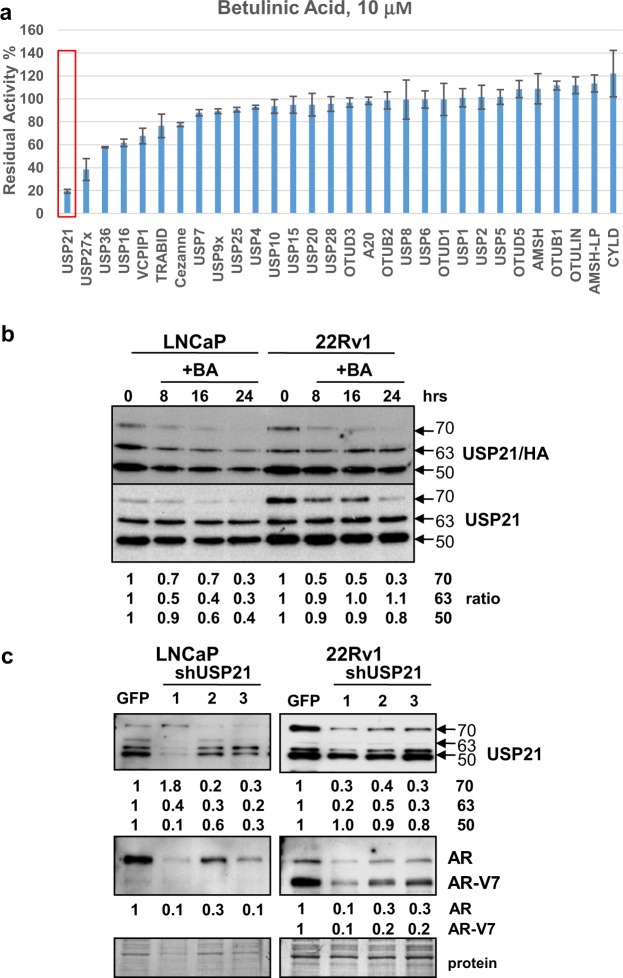
USP21 is a novel AR-regulatory DUB inhibited by BA. (**a**) MALDI-TOF analysis showed that 10 μM BA inhibited recUSP21 activity by 80% (red rectangle). BA also inhibited recUSP27 (60%), USP36 (40%), and USP16 (40%) to a lesser extent compared to USP21. Unlike results in LNCaP and 22Rv1 PCa cells (Fig. 6), BA had little effect on recUSP10 or 9X. (**b**) Western blot analysis of DUB labeling assay in LNCaP and 22Rv1 using HA-UbVS and USP21 specific antibody. Results showed that in LNCaP, BA inhibited 3 isoforms (70, 63, and 50 kDa) of USP21 activity (ratio of USP21/HA/total USP21 values for 3 isoforms shown below; 0 h = 1). In 22Rv1, BA inhibited the 70 kDa but not the 63 or 50 kDa isoforms of USP21. Blot images were cropped for clarity of the presentation. Similar results were obtained in an additional experiment. (**c**) Western blot showed that stable partial knockdown (2- to 10-fold) of USP21 isoforms in LNCaP and 22Rv1 with 3 different shRNAs (shUSP21-1, -2, -3) reduced AR and AR-V7 protein by 3- to 10-fold compared to shGFP controls. Loading control (protein). Blot images were cropped for clarity of the presentation.

We then determined if BA can directly inhibit recombinant (rec)DUB enzyme activity using matrix-assisted laser desorption/ionization time-of-flight (MALDI-TOF) mass spectrometry^47^. Using multiple recDUBs, the results demonstrated that 10 μM BA specifically inhibited recUSP21 activity by 80%, with an IC50 of 6.1 μM (Fig. 8a; Supplementary Fig. 3). BA also inhibited other recDUBs (USP27X, 16, 36), although to a lesser extent (40–60%). USP21 is an interesting candidate because it is proposed to be an H2A DUB^33–35^. Unlike the DUB labeling results in LNCaP and 22Rv1 PCa cells (Fig. 6), BA had little effect on recUSP10 or 9X activities, suggesting that other cofactors may be required inside cells.

DUB labeling with HA-UbVS showed that BA treatment of LNCaP and 22Rv1 cells inhibited active USP21 (Fig. 8b). Since USP21 has not previously been shown to regulate AR expression, we further analyzed LNCaP and 22Rv1/shUSP21 stable knockdown cells using 3 different shRNAs. Results indicated that (1) the 3 main isoforms of USP21 (70, 63, and 50 kDa) were specific and (2) partial USP21 knockdown decreased AR and AR-V7 3–10-fold (Fig. 8c).

### WP1130, a known DUB inhibitor, also decreases AR and increases PCa-specific cell death

**Figure 9:**
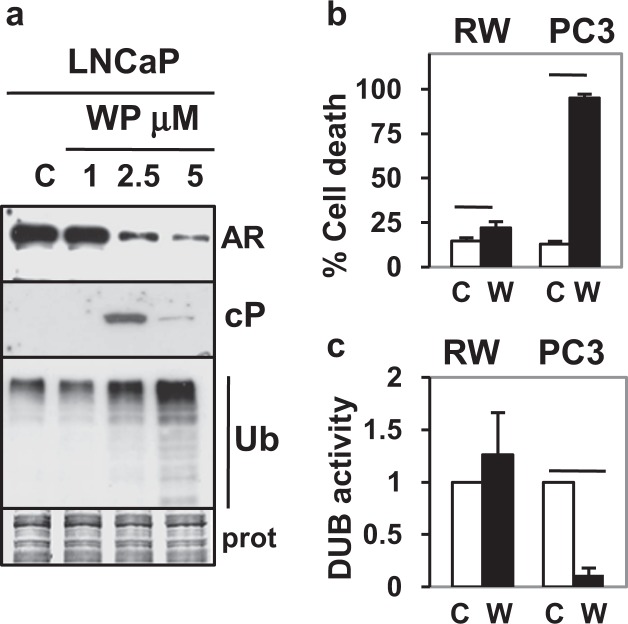
WP DUB inhibitor also decreases AR protein and has little effect in non-cancer cells. (**a**) Western blot analysis showed that WP (2.5 and 5 but not 1 μM; 16 h) decreased AR protein and increased cl-PARP (cP), poly-Ub in LNCaP. Loading control (prot). Blot images were cropped for clarity of the presentation. (**b**) Trypan blue assay showed that WP (W; 5 μM, 24 h) only slightly increased cell death in non-cancer RWPE-1 (RW) prostate epithelial cells compared to vehicle control (C) (*P* = 0.006) but greatly increased cell death in PC3 (*P* = 4 × 10^−9^). (**c**) DUB assay showed that WP (W; 5 μM, 24 h) did not inhibit DUB activity in RW but greatly inhibited DUB activity in PC3 (*P* = 0.002). In (**b**,**c**), n = 4–6, 2 independent experiments.

Our previous results suggest that the PCa-specific ability of BA to decrease AR and other pro-survival proteins and increase cell death may be due to inhibition of multiple DUBs in cancer but not in non-cancer cells^8^. However, it is not clear if BA’s PCa-specific effect is a unique feature of its anti-cancer capability independent of DUB inhibition or if other DUB inhibitors have a similar effect. WP1130 (WP) is a small molecule inhibitor of multiple DUBs (USP9X, 5, 14, UCHL5) that increases cl-PARP and poly-Ub accumulation in lymphoma cells and decreases the oncogenic transcription factor ERG, a key driver of PCa^27,48^. Our results showed that like BA, treatment of LNCaP cells with WP (2.5, 5 μM) potently induced cell death (50, 74%), decreased AR protein, and increased cl-PARP/poly-Ub (Fig. 9a; Supplementary Fig. 4). DUB labeling with HA-UbVS showed that WP (2.5 μM) treatment of LNCaP and 22Rv1 cells inhibited USP10, 21, and 9X activity but not USP7 (Supplementary Fig. 5). A summary of BA/WP inhibition of specific DUBs in LNCaP and 22Rv1 PCa cells and in MALDI-TOF mass spectrometry is presented in Supplementary Table 1. In non-cancer RWPE-1 prostate epithelial cells, WP only slightly increased cell death and did not inhibit DUB activity (Fig. 9b,c). In contrast, treatment of PC3 PCa cells with WP resulted in substantial cell death and decreased DUB activity. Since PC3 cells do not express AR, the WP and BA increase in cell death is independent of AR and likely requires inhibition of DUBs and poly-Ub accumulation. Overall, these results support the hypothesis that inhibition of multiple DUBs in PCa (either with BA or WP) reduces AR expression and increases cancer-specific cell death.

### AR antagonist Enz enhances BA AR degradation and cell death in CRPC

**Figure 10:**
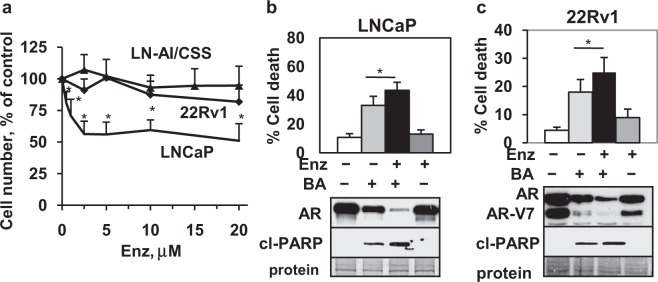
BA + Enz is a novel combination that decreases AR and increases cell death in PCa cells. (**a**) Cell viability assay (72 h) showed that LNCaP PCa but not LN-AI/CSS or 22Rv1 CRPC cells were sensitive to Enz (*P < 0.004; n = 9, 3 independent experiments). (**b,c**) Trypan blue exclusion (LNCaP, 16 h; 22Rv1, 24 h) and western blot (LNCaP, 24 h; 22Rv1, 16 h cl-PARP, 24 h AR) showed that BA (10 μM) + Enz (10 μM) further decreased AR and increased cell death and cl-PARP (cP) in LNCaP and 22Rv1 compared to BA alone (*P < 0.02; n = 6–9, 3 independent experiments). Loading control (protein). Blot images were cropped for clarity of the presentation.

Since it is likely that combinations of chemotherapeutic agents will be required for effective treatment of CRPC^1,2^, we determined whether combining BA with the FDA approved AR antagonist Enz is useful against PCa and CRPC cells, especially if resistant to Enz. Our results showed that LNCaP (androgen-dependent) were sensitive but 22Rv1 and LN-AI/CSS (CRPC) were resistant to Enz (0.5–20 μM) (Fig. 10a). Treatment of LNCaP, 22Rv1, and LN-AI/CSS cells with the BA + Enz combination further decreased AR protein and increased total cell death and cl-PARP compared to BA alone (Fig. 10b,c; Supplementary Fig. 6). Similar results were obtained with the WP + Enz combination in LNCaP, suggesting that Enz enhanced DUB inhibitor AR protein degradation and cell death (Supplementary Fig. 7). Overall, these results suggest that the BA or WP + Enz combination may provide a novel treatment strategy for PCa and CRPC by decreasing AR expression and increasing cell death.

## Discussion

**Figure 11:**
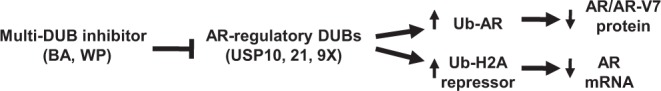
BA inhibits specific AR-regulatory DUBs to decrease AR protein and mRNA. Multi-DUB inhibitors BA and WP are proposed to inhibit AR-regulatory DUBs USP10, 21, and 9X in PCa cells to (1) increase poly Ub and Ub-AR/protein degradation and decrease AR/AR-V7 protein and (2) increase Ub-H2A repressor and decrease AR transcription (mRNA).

Our data is supportive of the hypothesis that inhibiting the activity of multiple and selective DUBs with BA or WP decreases AR/AR-V7 variant expression and kills PCa and CRPC cells without harming non-cancer cells^8^. It appears that the DUBs (e.g., USP10, 21) that regulate AR expression are also important in regulating AR-V7 variant. In addition to reducing AR protein, our data indicated that BA also decreased AR mRNA, making it a very attractive agent for the treatment of PCa and CRPC (Fig. 11). Our data also showed that the combination of BA or WP with the clinically approved AR antagonist Enz may be a novel treatment strategy for CRPC resistant to Enz by enhancing AR degradation and increasing apoptotic cell death. Therefore, we suggest that multiple selective DUBs are the main targets of BA and WP’s anti-AR and PCa apoptotic cell death effect.

BA has a remarkably broad efficacy against a variety of cancers, including cancers resistant to commonly used chemotherapeutic agents^6,7^. Selective reduction of multiple proteins important in proliferation, apoptosis, angiogenesis, and inflammation is a common feature for BA treatment of cancer cells as demonstrated by its effects on cyclins, Bcl-2, survivin, XIAP, AKT, Sp transcription factors, VEGF, IκBα, and topoisomerase^6–8,38,49^. BA is known to target the mitochondria by inducing apoptotic and necrotic cell death due to its effects on multiple Bcl-2 family members^6,7,36^. However, BA has diverse cancer cell context-dependent effects, which complicates the identification of a common mechanism upstream of the mitochondria. Based on our previously published report^8^ and data presented here, we hypothesize that the therapeutic effect of BA on cancer cells is due to inhibition of multiple and selective DUBs, which results in the reduction of specific pro-oncogenic proteins including AR and AR-V7 variant. Significantly, BA is also reported to have little or no cell death effects on normal cells and tissues, but the mechanism of this feature is currently unknown^8,50,51^. It has recently been shown that unlike in cancer, normal adult cells are more refractory to chemotherapeutic agents due to lower expression of apoptotic machinery proteins^52^. Similarly, we suggest that there may be differences in the expression of DUBs or their cofactors that sensitize cancer but not normal cells to BA induced cell death.

We suggest that BA possibly decreased AR mRNA by inhibiting an H2A DUB thereby increasing abundance of Ub-H2A, a known transcriptional repressor^33–35^ (Fig. 5). Our data using the MALDI-TOF DUB activity assay and multiple recDUBs indicated that BA directly inhibited recUSP21, one of the known H2A DUBs (Fig. 8a). In addition, BA also inhibited USP21 in LNCaP and 22Rv1 cells and a partial knockdown of USP21 resulted in a strong reduction of AR protein (Fig. 8b,c). A potential role for USP21 in the regulation of AR expression has not been previously reported and requires further investigation. BA is also reported to selectively degrade multiple members of the Sp transcription factor family (possibly by inhibiting Sp-regulatory DUBs), which is important in the transcriptional activation of AR^38,53,54^. Further work is required to determine if the decrease in AR mRNA by BA is due to inhibition of USP21, increase in Ub-H2A, and/or decrease in Sp promoter occupancy of the AR gene. Our results suggest that USP21 may be a novel AR-regulatory DUB and offer a new target for PCa therapy.

A potential reason why AR is more highly expressed in CRPC or after ADT may be the changes occurring in the expression or activity of AR-regulatory DUBs. It will be of interest to determine if higher expression of AR-regulatory DUBs in CRPC protects AR protein from UPS-mediated degradation and/or removes the transcriptional block on AR mRNA expression. Interestingly, USP22, an AR-regulatory DUB, is more highly expressed in CRPC and is one of the genes associated with a death from cancer signature^21^. In addition to USP22, USP7 and 12 have also been reported to be more highly expressed in PCa compared to non-cancer tissue by IHC^20,22^. Although our data suggests that BA and WP reduced AR expression in PCa by inhibiting multiple DUBs, we do not yet know with certainty the identity of all the specific DUBs inhibited by BA except for USP10, 21, and 9X. More conclusive evidence is required to identify additional AR-regulatory DUBs inhibited by BA or WP. In addition, since stable knockdown or overexpression of USP10 resulted in modest changes in AR protein (Fig. 7a,b), it is likely there are additional AR-regulatory DUBs inhibited by BA or WP, perhaps USP21 or 9X. Using a human PCa tissue microarray, our IHC data demonstrated that USP10 (AR-regulatory DUB) was strongly expressed in normal prostate but had greater variability with increasing Gleason grades (Fig. 7c). This result contradicts the idea that higher expression of AR in CRPC correlates with AR-regulatory DUBs. However, there is evidence that AR expression in human PCa tissue is heterogeneous, especially in metastatic CRPC^55–57^. Whether AR-regulatory DUBs are co-expressed with AR expressing cells in PCa tissue remains to be determined.

There is substantial evidence that resistance to Enz is due to changes in AR and increased expression of AR variants^40,42,58,59^. Given that BA reduced both full-length AR and AR-V7 variant expression (Fig. 2), there is rationale for its use in combination with Enz. Support for this strategy was our data showing that treatment of LNCaP PCa (sensitive to Enz) and 22Rv1/LN-AI/CSS CRPC cells (resistant to Enz) with the BA + Enz combination further decreased AR protein and increased apoptotic cell death compared to BA alone (Fig. 10; Supplementary Fig. 6). The WP + Enz combination also decreased AR and increased cl-PARP in LNCaP cells (Supplementary Fig. 7) suggesting that multi-DUB inhibitors may enhance the efficacy of Enz. Whether the *in vitro* efficacy of BA or WP + Enz (or other ADT) will replicate in animal models of CRPC is worth investigation. To begin to address the concern that BA and WP’s low solubility will have limitations for clinical use, the therapeutic value of a new orally bioavailable BA formulation and a more soluble analog of WP have been investigated in preclinical mouse models^32,60^.

There is a great emphasis on discovering newer AR antagonists or new agents that can specifically degrade AR and its variants with the expectation that Enz resistant CRPC will respond^61,62^. However, like Enz, it is probable that these new AR-specific agents will not be effective in a subset of CRPC patients or rapidly develop resistance. A major advantage of using multi-DUB inhibitors such as BA or WP is that neither agent requires AR for efficacy and they can target multiple pro-survival and proliferation genes. Since AR expression is heterogeneous especially in metastatic CRPC^55–57^, inclusion of agents such as BA or WP that also kill AR negative CRPC cells will likely have a positive role in preventing disease recurrence with less toxicity to normal cells.

## Methods

### Reagents

BA and WP were obtained from Biovision (Milpitas, CA); QVD from R & D Systems (Minneapolis, MN); CsA and SP600125 from Enzo Life Sciences (Farmingdale, NY); Chx from Sigma-Aldrich (St. Louis, MO); Btz and U0126 from LC Laboratories (Woburn, MA); LY294002 from EMD Millipore (Billerica, MA); HA-UbVS from Boston Biochem (Cambridge, MA); Enz from Selleckchem (Houston, TX); Coomassie blue from EMD Biosciences (Temecula, CA); and trypan blue (0.4%) from Invitrogen (Carlsbad, CA). All other reagents were purchased from Sigma-Aldrich.

### Cell culture

Human PCa cell lines LNCaP, DU145, PC3, and 22Rv1^43,63^ were obtained from the American Type Culture Collection (ATCC) and used within 6 months of resuscitation of original cultures. Unlike LNCaP, LN-AI cells can grow for long-term in RPMI 1640 with 5% charcoal-stripped fetal bovine serum (CSS; Hyclone, Logan, UT) and are referred to as LN-AI/CSS^64^. All other PCa cells were maintained in RPMI 1640 medium (Invitrogen, Carlsbad, CA) with 5% fetal bovine serum (Hyclone), 100 U/ml penicillin, 100 μg/ml streptomycin, and 0.25 μg/ml amphotericin (Invitrogen). Media for viral transduced LNCaP and 22Rv1 cells also contained puromycin (2 μg/ml; Invitrogen). RWPE-1 normal prostate epithelial cells were obtained from ATCC and maintained in Keratinocyte-SFM media (Invitrogen)^65^.

### Drug treatments

PCa cells were cultured in media containing BA (10 μM) +/− CsA (10 μM), QVD (10 μM), Chx (25 μg/ml), Btz (5 nM), LY294002 (20 μM), U0126 (10 μM), SP600125 (20 μM), Enz (10 μM), or DMSO (0.1–0.25%) control for varying times (24–72 h). WP was used at 1, 2.5, and 5 μM or WP (2.5 μM) + Enz (10 μM) for 16 h. In all the experiments, floating and trypsinized attached cells were pooled for further analysis.

### Trypan blue exclusion assay to measure total cell death

Treated and control cells were harvested, resuspended in PBS, diluted 1:1 in 0.4% trypan blue, dead blue and live non-blue cells immediately counted using a hemocytometer, and the % dead blue cells determined from at least three independent experiments done in duplicate.

### Western blot analysis

Preparation of total protein lysates and western blot analysis was done as previously described^64^. The following antibodies were used: cl-PARP (9541), P-AKT (Ser473; 587F11), AKT (9272), P-ERK1/2 (Thr202/Tyr204; 9101), ERK1/2 (9102), P-JNK (Thr183/Tyr185; 9251), Ub-H2A (Lys119) (D27C4) from Cell Signaling Technology (Danvers, MA); AR (N-20, 441), Ub (P4D1), JNK1 (FL), actin (C-11), horseradish peroxidase-conjugated secondary antibody from Santa Cruz Biotechnology (Santa Cruz, CA); USP7 (A300-033A), USP9X (A301-350A), USP10 (A300-900A) from Bethyl Laboratories (Montgomery, TX); and USP21 (HPA028397) from Sigma-Aldrich). Precision Plus Protein Dual Color Standards (Bio-Rad Laboratories, Hercules, CA) was used to estimate molecular weights in kDa. After immunodetection, our preference for loading controls was staining of total proteins transferred to the membrane with Coomassie blue because drug treatments often affect the levels of typical housekeeping proteins such as actin or tubulin. Blot images were cropped for clarity of the presentation. Full uncropped blot images are available upon request.

### Immunoprecipitation (IP) and immunoblot (IB)

LNCaP and 22Rv1 cells were treated with BA (10 μM) and control (0.25% DMSO) for 18–20 h and cell pellets immediately lysed in NP40 lysis buffer (same used for western blot)^64^. AR (441; 0.5 μg) or Ub (1 μg) antibody was added to 400 μg of total protein and incubated 4 °C overnight with rotation. Negative control IP was addition of an equal amount of non-specific mouse Ig (Santa Cruz). Protein A/G agarose (20 μl; Santa Cruz; pre-washed with NP40 buffer) was added for 2 h, 4 °C with rotation followed by centrifugation, washing pellets 3x with PBS, and final pellet resuspended in SDS loading buffer for analysis by western blot (Ub, AR IB). Total protein lysates were run on a separate gel.

### Quantitative Real Time Polymerase Chain Reaction (qPCR)

The procedure for qPCR was done as previously described^49^. The following DNA oligonucleotides from Eurofins MWG Operon (Huntsville, AL) were used for qPCR: AR sense 5′-GACGACCAGATGGCTGTCATT-3′ and antisense 5′-GGGCGAAGTAGAGCATCCT-3′ (106 amplicon) (Primer Bank)^66^. Values from two independent experiments done in duplicate were normalized to the housekeeping reference genes ribosomal protein, large, P0 (RPL0) sense 5′-GCAATGTTGCCAGTGTCTG-3′ and antisense 5′-GCCTTGACCTTTTCAGCAA-3′ (141 amplicon) and β-2 micoglobulin sense 5′-ATGAGTATGCCTGCCGTGTGA-3′ and antisense 5-GGCATCTTCAAACCTCCATG-3′ (101 amplicon)^67^.

### Immunofluorescence

Adherent and non-adherent LNCaP cells were harvested after a 24 h treatment with BA (10 μM) or control, applied to slides by smearing, air dried, fixed in formalin for 10 min, permeabilized with 0.1% Triton X-100/PBS for 10 min, rinsed with water, and blocked with goat serum (Vector Laboratories, Burlingame, CA) for 30 min. Ub-H2A was immunostained using Ub-H2A (Lys119) (D27C4) (Cell Signaling) at 1:50 dilution for 1 h followed by secondary antibody Alexa Fluor 488 (green) goat anti-mouse IgG (1/500 dilution; Invitrogen) for 1 h. Mounting medium with DAPI was from Vector Laboratories. Color images were acquired using a Nikon Eclipse 90i fluorescence microscope and merged using Adobe Photoshop 7.

### IHC of Ub-H2A in TRAMP PCa

We obtained paraffin sections from TRAMP mice with PCa treated with BA (10 mg/kg body weight) or vehicle control from our previous study^8^. Immunostaining for Ub-H2A (D27C4) (Cell Signaling) was performed using a 1/200 dilution of mouse monoclonal and the MOM Peroxidase Kit (Vector Laboratories) following the manufacturer’s instructions and as we previously described^68^.

### Human PCa tissue microarray and USP10 IHC

Human PCa tissue microarray PR803a was purchased from US Biomax, Inc. (Rockville, MD) and utilized for immunostaining of USP10 (A300-900A at 1/50 dilution from Bethyl Laboratories; verified for IHC) using the methods previously described^68^. The slide contains unstained paraffin sections of 64 cases of PCa categorized as Gleason grade 4–6 (n = 12), 7 (n = 23), and 8–10 (n = 29), and normal prostate tissue (n = 4).

### DUB labeling and activity assays in PCa cells

LNCaP and 22Rv1 cells treated with BA (10 μM) or control (0, 8, 16, 24 h) were harvested, lysed in DUB buffer (50 mM Tris-HCl, pH 7.5, 0.1% NP-40, 5 mM MgCl_2_, 250 mM sucrose, 1 mM DTT, 1 mM PMSF), centrifuged, 25 µg of protein incubated with 0.5 μg HA-UbVS for 1.5 h at room temperature, and samples analyzed by western blot using anti-USP10, 21, 9X, and 7 to detect DUB activity (USP/HA). Lysates without HA-UbVS were also analyzed by western blot to detect effect on total USP10, 21, 9X, and 7. Quantitative differences for USP/HA and total USP were determined by measuring pixel intensity from images (UNSCAN-IT; Silk Scientific, Inc., Orem, UT), normalization to Coomassie blue stained total protein, and the ratio of USP/HA/total USP determined (control 0 h = 1). A similar analysis was done with LNCaP and 22Rv1 cells treated with WP (2.5 μM) or control (0, 4, 8, 16 h). DUB-Glo Protease Assay (Promega, Madison, WI) was used to determine the effect of WP (5 μM) on DUB activity in confluent RWPE-1 and PC3 cells treated with WP or control for 24 h as previously described^8^. Light units (control treatment = 1) from 4 samples were determined from two independent experiments.

### BA + recDUB assay by MALDI-TOF mass spectrometry

Methods were as previously described^47,69^. In brief, recDUBs (see Fig. 8a) were freshly prepared in the reaction buffer (40 mM Tris–HCl, pH 7.6, 5 mM DTT, 0.005% BSA), pre-incubated with BA (10 μM) for 15 min at 30 °C, diubiquitin isomers added, incubated for 60 min at 30 °C, and the reaction stopped with trifluoroacetic acid (2% final). Acidified samples of the DUB assays were mixed with 0.5 mM ^15^N-ubiquitin and one part of 2,5 DHAP matrix solution and 0.2 μl spotted in replicates onto an MTP AnchorChip 1,536. We then determined BA’s IC50 on recUSP21 as previously described^47^.

### USP 10 and 21 viral transduction of LNCaP and 22Rv1

The shRNA design, lentivirus production, and infection were done as previously described^70^. The following DNA oligonucleotides (Eurofins MWG Operon) targeting USP10 were cloned into pLKO.1 lentivirus vector: shUSP10-1 sense: 5′-CCGGGCCTCTCTTTAGTGGCTCTTTCTCGAGAAAGAGCCACT AAAGAGAGGCTTTTTG-3′; shUSP10-1 antisense: 5′-AATTCAAAAAGCCTCTCTTTAGTGGC TCTTTCTCGAGAAAGAGCCACTAAAGAGAGGC-3′; shUSP10-2 sense: 5′-CCGGCCTATGTG GAAACTAAGTATTCTCGAGAATACTTAGTTTCCACATAGGTTTTTG-3′; shUSP10-2 anti- sense: 5′-AATTCAAAAACCTATGTGGAAACTAAGTATTCTCGAGAATACTTAGTTTCCACATA GG-3′; shUSP10-3 sense: 5′-CCGGCCCATGATAGACAGCTTTGTTCTCGAGAACAAAGCTG TCTATCATGGGTTTTTG-3′; shUSP10-3 antisense: 5′-AATTCAAAAACCCATGATAGACAGC TTTGTTCTCGAGAACAAAGCTGTCTATCATGGG-3′. The following DNA oligonucleotides (Eurofins MWG Operon) targeting USP21 were cloned into pLKO.1 lentivirus vector: shUSP21-1 sense: 5′-CCGGGCCTTTCTACTCTGATGACAACTCGAGTTGTCATCAGAGTAGAAAGGCTT TTTG-3′; shUSP21-1 antisense: 5′-AATTCAAAAAGCCTTTCTACTCTGATGACAACTCGAGTT GTCATCAGAGTAGAAAGGC-3′; shUSP21-2 sense: 5′-CCGGGACCCTCTGCAATATCACTTT CTCGAGAAAGTGATATTGCAGAGGGTCTTTTTG-3′; shUSP21-2 antisense: 5′-AATTCAAAA AGACCCTCTGCAATATCACTTTCTCGAGAAAGTGATATTGCAGAGGGTC-3′; shUSP21-3 sense: 5′-CCGGCCACTTTGAGACGTAGCACTTCTCGAGAAGTGCTACGTCTCAAAGTGGTT TTTG-3′; shUSP21-3 antisense: 5′-AATTCAAAAACCACTTTGAGACGTAGCACTTCTCGAGA AGTGCTACGTCTCAAAGTGG-3′. All DNA sequences were previously validated and obtained from Mission shRNA library (Sigma-Aldrich). The control shRNA was targeted against green fluorescent protein (GFP). Flag-HA-USP10 (22543) plasmid was purchased from Addgene (Cambridge, MA)^71^. Retrovirus production and infection were done as previously described^72^. We used LNCaP/EV (empty vector) as the negative control cells^72^. Differences in AR protein between shUSP10-1, -2, -3 and shUSP21-1, -2, -3 were compared to shGFP (LNCaP and 22Rv1) and between LNCaP/USP10 and LNCaP/EV were determined by western blot as previously described^73^.

### Enz cell viability assay

The CellTiter Aqueous colorimetric method from Promega was used to determine cell viability of LNCaP, 22Rv1, and LN-AI/CSS PCa cells in media containing Enz (0.5–20 µM) or control (0.1% DMSO). Cell viability was normalized against the DMSO control and the data expressed as a percentage of control from three independent experiments done in triplicate.

### Statistics

Statistical differences between drug-treated and control PCa cells were determined by two-tailed Student’s *t*-test (unequal variance) from 2–3 independent experiments done in duplicate or triplicate with *P* < 0.05 considered significant.

## Electronic supplementary material

Supplementary Figures 1-7 and Table 1

## Data Availability

Any resources resulting from this manuscript will be freely shared with other scientists upon request.
