# Supplementary material for: Inhibiting Multiple Deubiquitinases to Reduce Androgen Receptor Expression in Prostate Cancer Cells
Source: Sci Rep. 2018 Sep 3;8:13146. doi: 10.1038/s41598-018-31567-3 (PMC6120934; doi:10.1038/s41598-018-31567-3)
Supplement: Supplementary file 1 — Supplementary Figures 1-7 and Table 1 [file 41598_2018_31567_MOESM1_ESM.pdf]

Supplementary Figures 1-7
Supplementary Table 1

Inhibiting Multiple Deubiquitinases to Reduce Androgen Receptor Expression in Prostate Cancer Cells

Alicia de las Pozas, Teresita Reiner, Virginia De Cesare, Matthias Trost & Carlos Perez-Stable

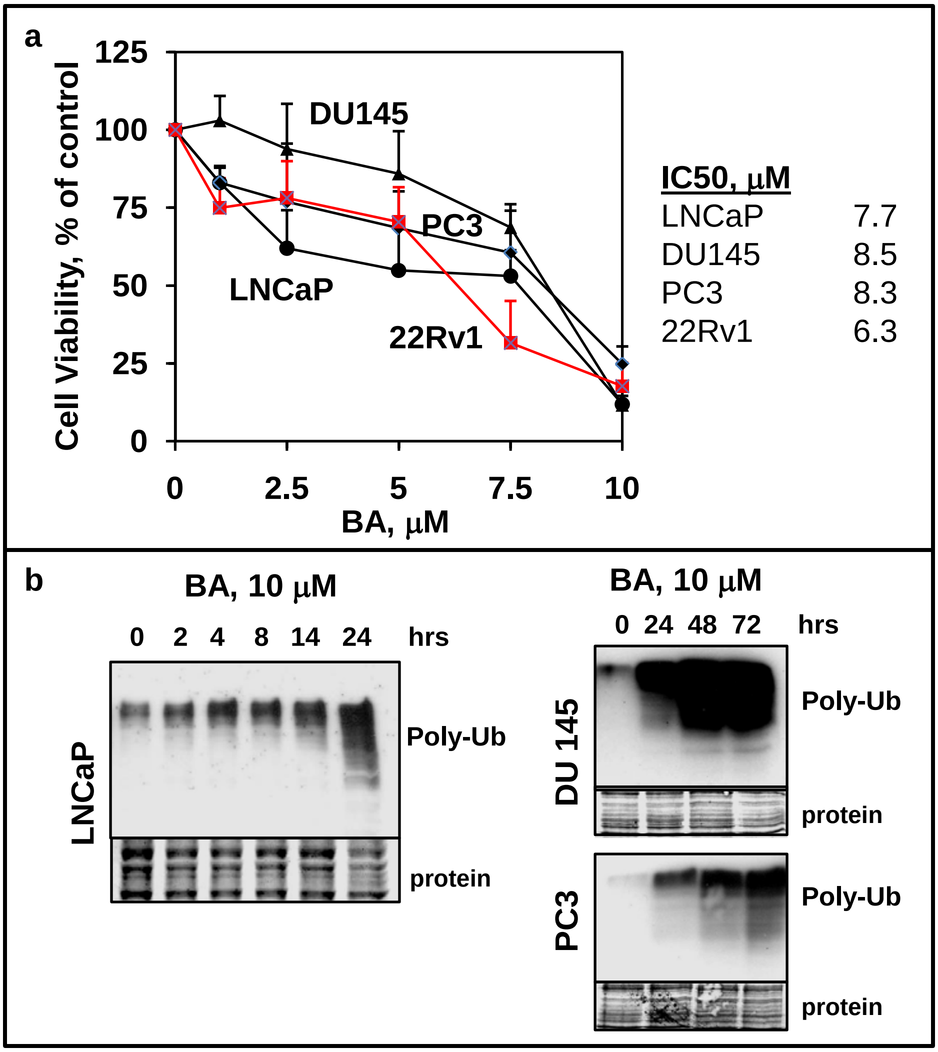

Supplementary Figure 1. BA inhibits proliferation and increases poly-Ub in multiple PCa cells. (**a**) Cell proliferation assay showed that increasing concentrations of BA (1.0-10 μM) inhibited LNCaP, DU145, PC3, and 22Rv1 (red line) cells. IC50 values are shown to the right. (**b**) Western blot showed that BA (10 μM) increased poly-Ub in LNCaP, DU145, and PC3 cells with increased time. Loading control (protein).

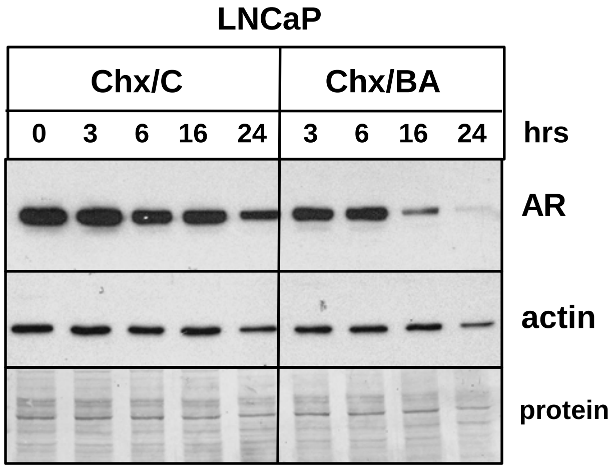

Supplementary Figure 2. BA decreases AR protein stability in LNCaP cells. Western blot showed that Chx (25 μg/ml) + BA (Chx/BA) decreased AR protein more rapidly especially at 16 and 24 h compared to Chx + control (Chx/C) in LNCaP cells. There were less effects on actin. Loading control (protein). Blot images were cropped for clarity of the presentation.

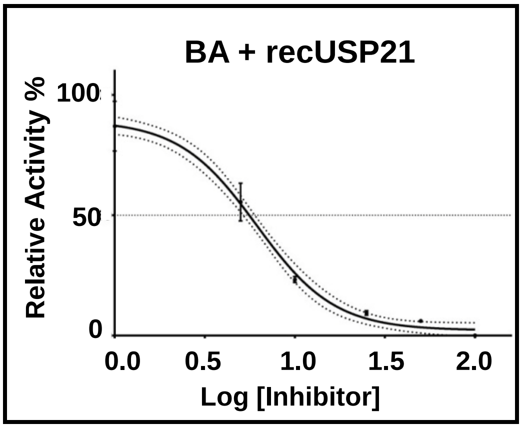

Supplementary Figure 3. MALDI-TOF analysis showed that BA specifically inhibited recUSP21 with an IC50 of 6.1 μM.

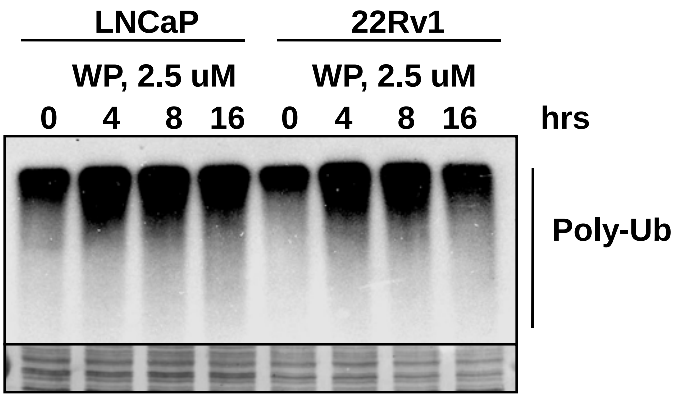

Supplementary Figure 4. Western blot showed that WP (2.5 μM) increased poly-Ub in LNCaP and 22Rv1 cells.

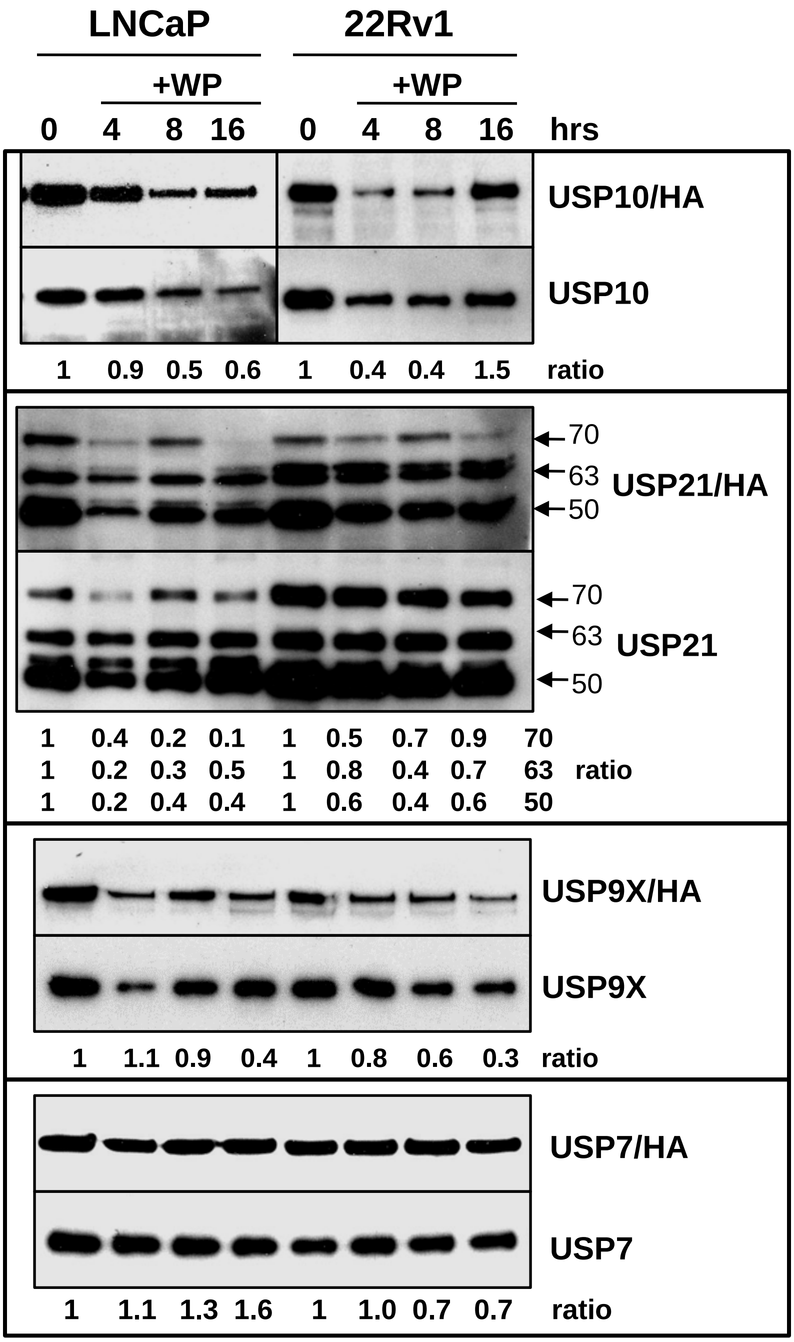

Supplementary Figure 5. WP inhibits USP10, 21, and 9X in LNCaP and 22Rv1 cells. Western blot analysis of DUB labeling assay using HA-UbVS and USP10, 21, 9X, and 7 specific antibodies. Results showed that WP (2.5 μM) inhibited USP10 activity (ratio of USP10/HA / total USP10 values shown below; 0 h = 1; vertical line indicates separate blots). WP also inhibited USP21 isoforms (70, 63, 50 kDa) and USP9X activity in both LNCaP and 22Rv1. There was little effect of WP on USP7 activity. Blot images were cropped for clarity of the presentation. Similar results were obtained in an additional experiment.

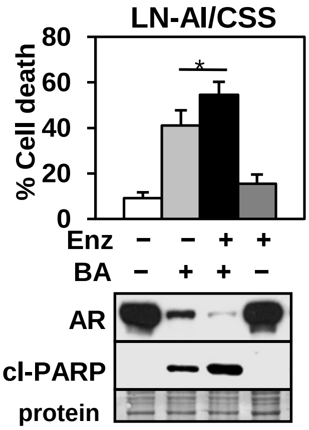

Supplementary Figure 6. BA + Enz combination decreases AR and increases cell death in LN-AI/CSS PCa cells. Trypan blue exclusion and western blot (48 h) showed that BA (10 μM) + Enz (10 μM) further decreased AR and increased cell death and cl-PARP compared to BA alone (*, $P<0.05$; n=4-6, 2 independent experiments). Loading control (protein). Blot images were cropped for clarity of the presentation.

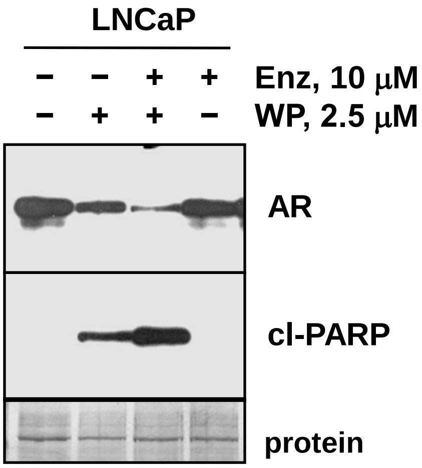

Supplementary Figure 7. WP + Enz decreases AR and increases cl-PARP. Western blot (16 h) showed that WP (2.5 μM) + Enz (10 μM) decreased AR and increased cl-PARP greater than WP alone in LNCaP cells. Loading control (protein). Blot images were cropped for clarity of the presentation.

DUB	LNCaP BA	LNCaP WP	22Rv1 BA	22Rv1 WP	MALDI-TOF BA
USP10	+	+	+	+	−
USP21 -70	+	+	+	+	+ recUSP21
-63	+	+	−	−	
-50	+	+	−	−	
USP9X	−	+	+	+	−
USP7	−	−	−	−	−

Supplementary Table 1. Summary of BA/WP inhibition of multiple DUBs in LNCaP and 22Rv1 PCa cells (DUB labeling) and BA inhibition of recDUBs (MALDI-TOF mass spectrometry). Effect on USP21 isoforms (70, 63, 50) is shown.
